# Mobile Game Design Guide to Improve Gaming Experience for the Middle-Aged and Older Adult Population: User-Centered Design Approach

**DOI:** 10.2196/24449

**Published:** 2021-05-20

**Authors:** Seyeon Lee, Hyunyoung Oh, Chung-Kon Shi, Young Yim Doh

**Affiliations:** 1 Graduate School of Culture Technology Korea Advanced Institute of Science and Technology Daejeon Republic of Korea

**Keywords:** mobile games, older adults, middle-aged adults, design guideline, gaming experience

## Abstract

**Background:**

The number of older adult gamers who play mobile games is growing worldwide. Earlier studies have reported that digital games provide cognitive, physical, and socioemotional benefits for older adults. However, current mobile games that understand older adults’ gameplay experience and reflect their needs are very scarce. Furthermore, studies that have analyzed older adults’ game experience in a holistic manner are rare.

**Objective:**

The purpose of this study was to suggest mobile game design guidelines for adults older than 50 years from a holistic gaming experience perspective. Adopting a human-centric approach, this study analyzes middle-aged and older adults’ gameplay experience and suggests practical design guides to increase accessibility and satisfaction.

**Methods:**

We organized a living laboratory project called the “Intergenerational Play Workshop.” In this workshop, 40 middle-aged and older adults (mean age 66.75 years, age range 50-85 years) played commercial mobile games of various genres with young adult partners for 1 month (8 sessions). Using a convergent parallel mixed-method design, we conducted a qualitative analysis of dialogue, game diaries, and behavioral observations during the workshop and a quantitative analysis of the satisfaction level of the game elements for the mobile games that they played.

**Results:**

This project was active from April 2019 to December 2021, and the data were collected at the workshops from July 1 to August 28, 2019. Based on the identified themes of positive and negative experiences from the qualitative data, we proposed 45 design guides under 3 categories: (1) cognitive and physical elements, (2) psychological and socioemotional elements, and (3) consumption contextual elements. Our empirical research could reaffirm the proposals from previous studies and provide new guidelines for improving the game design. In addition, we demonstrate how existing commercial games can be evaluated quantitatively by using the satisfaction level of each game’s elements and overall satisfaction level.

**Conclusions:**

The final guidelines were presented to game designers to easily find related information and enhance the overall understanding of the game experience of middle-aged and older adults.

## Introduction

### Background

The number of mobile game players has increased annually to 1.1 billion in 2017 and 1.5 billion in 2020. It is expected to increase by 50 million to 100 million people every year and to reach 1.8 billion by 2024 [1]. The rise of smartphone penetration [2] could give older adults an opportunity to easily access digital games. According to the American Association of Retired Persons research in 2019, 73% of game players aged 50 years and older use mobile devices in the United States—this proportion has increased from 57% in 2016, which was only a few years earlier. In contrast, the proportion of game players using computers and laptops decreased from 56% in 2016 to 47% in 2019 [3]. The Korean mobile game market for the older adult population is developing actively with high smartphone penetration rates and high Wi-Fi accessibility in public places. In a survey conducted in 2020, 52.1% of Koreans in their 50s and 31.9% of Koreans aged 60 to 65 years mentioned that they played mobile games [4]. Previous studies have reported various benefits of digital gameplay for older adults, including lower cognitive decline [5-7], increased physical activity [8-11], and improved socioemotional status [12-14]. However, most digital games cater to the younger generations, making it difficult for many older adults to play them [15,16]. In addition, game designers may not be sensitive to the gaming experience of older adults because most of them belong to the younger generation. Therefore, it is necessary to improve the game design based on the understanding of older adults’ needs, preference, and play experience. As the demand for mobile games is expected to increase among middle-aged and older adults in the future, this study aims to provide experience analysis and design guidelines focused on mobile games.

### Design Guide for Age-Related Changes

Earlier studies have reported various factors that cause difficulties for older adults to play mobile games. In a survey of Canadian gamers aged over 55 years, the highest ranked difficulty was that the games were too complicated [17]. In addition, age-related limitations such as declining vision, hearing, cognition, and motor functions can cause difficulties in playing games [18]. Older adults who were skilled gamers did not experience many difficulties, but they complained of difficulty in changing game settings, small object size, time limit, understanding rules, and fast pace [16]. To improve the accessibility of digital technologies such as computers and the internet, many studies have suggested interface design guides for older adults, considering age-related changes such as vision, motor control, hearing, cognition, and so on [19-21]. Several attempts have been made to apply a similar approach to digital game design. Gamberini et al [22] studied age-related changes in terms of perception (vision, hearing, and touch/movement), attention, learning/memory, and cognitive tasks and proposed specific implications for game design. Fua et al [23] also studied the relationship between cognitive abilities and gameplay mechanics for older adults, especially within populations at risk of dementia. In addition, Ijsselsteijn et al [24] provided insights into digital game design based on usability guides for older adults. Gerling et al [25] created structural models of gameplay and proposed guides that consider age-related changes under the categories of game elements, such as players and resources, user interface, core mechanics, and outcomes. However, these studies did not analyze the game experience of older adults through an empirical study. De Barros et al [26] improved their mobile game app by reflecting on older adults’ behavior and feedback, and they proposed recommendations under 3 categories, that is, navigation, interaction, and visual design.

### Design Guide Related to Psychological and Socioemotional Aspects

Various surveys conducted in the United States, Canada, South Korea, and Flanders showed similar results regarding game preferences of older adults. They indicated that older adults strongly prefer puzzles, card/board games, and strategy games to other genres. These types of games are mostly casual and easy to learn and play, but are also challenging [4,12,27,28]. Other studies also showed that older adults prefer an intellectual challenge to fighting and racing games, which require skills related to speed and reflexes [29,30]. Another experimental study identified how the enjoyment of older adults differs depending on the genre of the games. It was found that puzzle games were easier to play and more enjoyable than simulation games or action games [31]. In addition, older adults’ preference for the game genre did not differ between gamers and nongamers [32]. Among the 6 motives of game play presented from the uses and gratifications perspective [33], challenge was the highest among older adults, followed by arousal, diversion, fantasy, competition, and social interaction [27]. In addition, older adults played digital games for relaxation, fun, and to pass time [16]. For older adults, the emphasis was not only on fun associated with games but also on personal growth and benefits as motivations to play [34]. In fact, many older adults described playing games as a means of exercising their brain [35].

De Carvalho and Ishitani [36] conducted an empirical study to test existing mobile puzzle games in older adults and proposed guidelines related to motivational aspects for serious games. Cota et al [37] also found the main features that motivate the older adults by analyzing their evaluations of mobile games developed by the researchers. Both studies proposed design guides to encourage motivation, such as usability heuristics, clear and motivational feedback, clarifying the benefits of gaming, cognitive training, and providing proper difficulty levels.

Older adults play games mostly alone rather than with others [27,28]. However, baby boomers are community-oriented, and social interaction is an important motivation for them [30]. Moreover, experimental studies have shown that playing with a partner leads to a higher enjoyment than playing with a computer [38,39]. In particular, middle-aged adults (40-59 years) are more likely to play games with their children [28]. In the United States, 57% of parents play games with their children at least once a week [40]. De Schutter and Vanden Abeele [41] recommended a game design that considered the psychosocial context of older adult players, which include finding the right playing partner, vicarious play, time management, sharing high scores, and balancing teams. Additionally, McLaughlin et al [42] conducted a qualitative study on the experience of older adults in terms of costs (eg, initial frustration, stereotype threat, emotional arousal, usability challenges) and benefits (eg, self-esteem, physical activity, social interaction, well-being, fun, learning). Marston [43] also analyzed older adults’ game experience empirically under a framework of 3 categories (older adults, technology, and content/interaction) and applied them to suggest design guidelines.

### Research Questions in This Study

While many user experience studies have been conducted on digital apps for older adults, not many have considered “playability” for older adults on “mobile game” media. Furthermore, studies that have analyzed older adults’ gaming experience in a holistic manner are rare. Some studies have investigated the elements of age-related changes and suggested guides [23-25]. However, they were not empirical studies. Some empirical studies have analyzed the experience of older adults; however, they lacked suggestions for various game elements under the structural framework [36,37,42]. In addition, previous guides did not include consumer behavior contexts related to mobile gameplay, such as devices, installation, in-game payment, and advertising. Moreover, some empirical studies suggested guides under the structural framework, but they tested only limited devices or genres [43]. Therefore, the purpose of this study was to suggest practical design guides based on empirical studies for game designers, including various mobile game elements, from the holistic perspective of middle-aged and older adults’ gaming experience. Game elements can be considered as a set of building blocks or features shared by games [44]. We used “game elements” in a broad interpretation to connect user-perceived game elements to game design elements. This study was guided by the following 3 research questions (RQs):

RQ1. What are the positive and negative experiences of middle-aged and older adults when playing mobile games?

RQ2. What mobile game design guides should be considered to embrace middle-aged and older adults?

RQ3. How satisfied are middle-aged and older adults with the game elements of commercial mobile games?

We planned to identify several themes of the gaming experience, design guidelines from qualitative analyses (RQ1 and RQ2), and provide useful indicators by presenting the satisfaction levels of the game elements in existing commercial games by using quantitative analysis (RQ3). This convergent parallel mixed-methods design [45] was used to identify important criteria that could affect the gaming experience of middle-aged and older adults and to determine how existing games can be evaluated based on these criteria.

## Methods

### Participants

We adopted a human-centered approach by using a living laboratory that promotes active involvement of users and applies their experience and feedback to the research [46]. We collaborated with 2 institutions that organize programs for middle-aged and older adults. One institution is the 50 plus campus that provides educational programs targeting adults in their 50s and 60s (middle-aged adult group) and the other institution is a senior welfare center targeting adults aged 65 or older (older adult group). We designed living laboratory activities where middle-aged and older adults played various genres of mobile games with young adult partners. During these activities, participants not only played games but also discussed their playing experiences and provided recommendations to improve game design. A total of 40 middle-aged and older adults participated, and Table 1 shows the demographic information of the participants.

**Table 1:** Demographic information of the participants (N=40).

Characteristics			Values
Age (years), mean (SD)			66.75 (9.31)
**Age range (years), n (%)**			
	50-59	11 (28)	
	60-69	14 (35)	
	70-79	12 (30)	
	80-85	3 (8)	
**Location, n (%)**			
	50 plus campus (middle-aged adult group)	20 (50)	
	Senior welfare center (older adult group)	20 (50)	
**Gender, n (%)**			
	Female	30 (75)	
	Male	10 (25)	
**Job status, n (%)**			
	Employed	12 (30)	
	Retired/homemaker	28 (70)	
**Education, n (%)**			
	Elementary/middle school	13 (33)	
	High school	7 (18)	
	Undergraduate school	12 (30)	
	Graduate school	8 (20)	
**Monthly income^a^, n (%)**			
	Low: Less than ₩2 million	18 (45)	
	Middle: ₩2-8 million	16 (40)	
	High: More than ₩8 million	5 (13)	
	No response	1 (3)	

^a^US $1=₩1200.

The recruitment process was conducted differently in both the places. Web-based recruitment was conducted at the 50 plus campus for a month, and 20 participants were recruited. Our orientation session explained the workshop activities and received informed consent forms for participation. Two participants did not attend the orientation, and one decided not to participate after the orientation. Therefore, we recruited 3 more participants. In the senior welfare center, offline recruitment was conducted for 1 month. Two social workers familiar with the members of the center participated in the recruitment and selected 20 mentally and physically healthy participants. We also held an orientation session and received informed consent forms. The recruitment results showed a high percentage of female participants. It can be assumed that this was because female members attending these 2 institutes tended to prefer new and coactivity programs over male members. 

Ten participants were grouped in 1 class, and there were 4 classes in the workshops (2 middle-aged groups and 2 older adult groups). Over 8 sessions, the number of absent days per participant was 0-4 times (average attendance rate, 91.6% [293/320]). However, no participant dropped out during the workshops in both places. Ethical approval was obtained from the institutional review board of the authors’ institute. Table 2 shows the prior gaming experience of the participants. There were 13 older adult participants who reported as currently playing games because they had participated in an earlier program that included serious games for cognitive training at the senior welfare center. The age of the first gaming experience was widespread among the middle-aged adult group, and most of the older adult participants started playing games at an older age.

**Table 2:** Prior gaming experience of the participants.

Characteristic		Values, n (%)
**Game experience (n=40)**		
	No gaming experience	12 (30)
	Currently gaming	15 (38)
	Have experience but no longer play	13 (33)
**Age of first playing games (years)^a^, (n=28)**		
	10-19	3 (11)
	20-29	3 (11)
	30-39	5 (18)
	40-49	2 (7)
	50-59	6 (21)
	More than 60	9 (32)

^a^Only for participants with gaming experience.

In addition, 11 undergraduate students (9 females and 2 males, mean age 21.45 years) majoring in the Department of the Senior Industry at Kangnam University participated in the living laboratory activities as supporting partners. In each class, 1 young partner was paired with 1 older participant. Young partners participated in all 4 classes and supported a total of 4 older participants per person. The other young partners assisted in the workshop activities. We also held an orientation session for young adult partners to receive their informed consent for participation and provide instructions for the role of the supporting partners. They helped older participants play games, played games together, and received feedback from them through questions.

### Games Played in the Workshops

Four graduate students studying game design and human-computer interaction developed the first list of games, which are popular and enjoyed across all age groups. They referred to the annual report of the popular game list and personal experience of playing with older adults. Each student added 20-60 game titles in various genres and platforms and scored the difficulty level of gameplay from 1 to 4 according to the time required to learn them (1: 10 minutes, 2: 1-2 hours, 3: a week, 4: more than a week). A total of 146 games were included in the list, and 3 authors finally selected 9 games to play during the workshops. Table 3 shows the games played in the workshops.

**Table 3:** Games played in the workshops.

Title		Genre		Game description
**Game played by both groups (age range, 50-85 years**)				
	Homescapes	Puzzle and story	A match-3 puzzle where the player solves puzzles presented with stories and decorates a house [47].	
	Fruit Ninja	Action	A fruit-slicing action game where the player swipes and slices fruits with a blade [48].	
	Long Journey of Life	Adventure and story	A story-based game where the player controls a small boat and sails through life stages from birth to death [49].	
**Games played by the middle-aged group only (age range, 50-64 years**)				
	Brawl Stars	Action	A multiplayer web-based battle arena game where players attack other players with the same team members [50].	
	Sally’s Law	Action and story	A platformer action game with a story and puzzle [51].	
	Good Pizza, Great Pizza	Management	The player becomes the owner of a pizza restaurant and makes pizzas according to orders [52].	
**Games played by the older adult group only (age range, 65-85 years**)				
	Word Tower-World Trip	Puzzle	A word puzzle game where the player swipes and connects syllables and finds hidden words [53].	
	Go-stop Plus	Web board	A web-based version of a Korean traditional card game that uses a deck of 48 cards [54].	
	Lonely One: Hole-in-one	Shooting and sports	A golf-based game where the player adjusts the parabola and releases to hit the ball [55].	

The following guidelines were used to choose the games. First, the game should be accessible in both mobile phones and tablet personal computers; 72 games were selected in this stage. Second, the game should be in Korean language; 64 games were further selected in this stage. Third, to ensure that the games were not too complicated for older adults, the difficulty level should be 1 or 2. In this stage, 54 games were selected from the remaining ones. There were 21 puzzle games, 6 action games, 6 web board games, 4 adventure games, 10 management/simulation games, 6 shooting games, and 1 rhythm game. Before we finalized the list, we had discussions with a social worker and a program manager in each collaborating institution regarding participant groups’ digital skills [56] in using mobile apps. In addition, we had a pilot session to test the games with 13 participants from the 50 plus campus. Finally, we selected 1-3 games from each genre in order to have an even distribution from a variety of genres such as puzzle, action, web board, adventure, and management games. Considering the digital skills of each participant group, we selected 3 common games for both groups and 3 different games for each group. “Go-stop Plus” and “Lonely One: Hole-in-one” were selected for the older adults’ group because they are based on card games and sports, which are familiar to older adults in real life. Participants also played other games not in this list according to their preferences during the living laboratory activities and provided their feedbacks.

### Workshop Procedure

**Figure 1:**
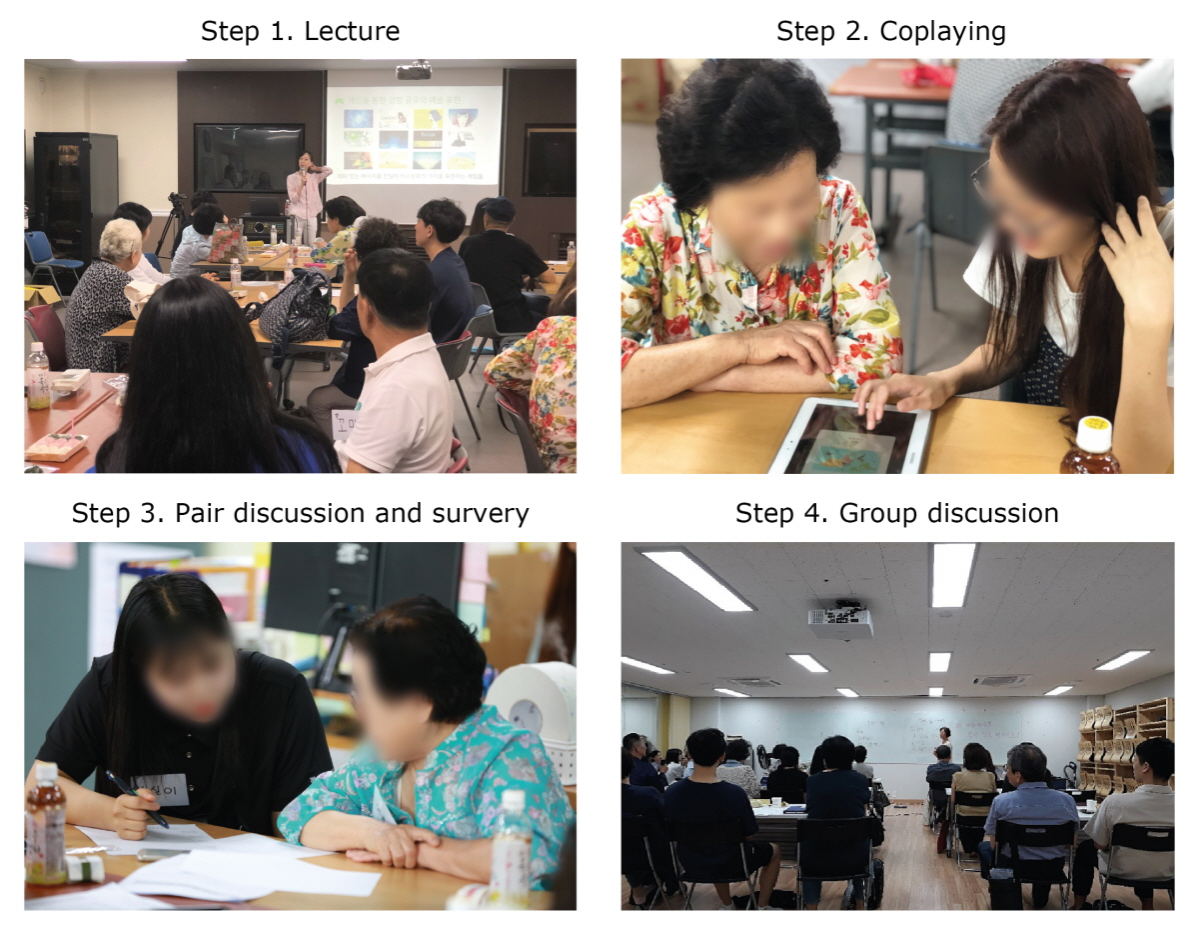
Photographs from the intergenerational play workshop.

We organized intergenerational play workshops twice a week for 4 weeks (8 sessions), and each session lasted 1-2 hours. Each session consisted of a short lecture (10-30 min), coplaying (20-40 min), survey and pair discussion (10-20 min), and group discussion (10-20 min). Figure 1 shows the workshop process.

The lectures dealt with how to play specific games and useful information to enhance the game literacy of those who are not familiar with digital games. After a short lecture, participants and young adult partners were paired, and they played games together. Each pair was provided a 10-inch tablet personal computer, but they could use their personal smartphone if they wanted. Young adult partners not only played games together but also taught participants how to play and get acquainted with the games. After playing a game, the young partner asked, “what were you satisfied with or unsatisfied with during the game play?”, to which the participants provided their responses. In addition, participants filled out the survey form by checking the satisfaction level for each element of the game. They discussed their gameplay experience and shared ideas of design recommendation during group discussions. Participants were compensated with ₩10,000 (approximately US $8) per session for participating in the workshops.

For over one month of the living laboratory activities, older adult participants could visit the play room at the senior welfare center and use the tablet personal computers, which had the recommended games installed. There was no playroom at the 50 plus campus, but middle-aged adult participants could borrow the tablet personal computers and play games at home. Both groups could also install and play games on their personal mobile phones. Researchers opened a web-based community and asked participants to upload diaries with their experience after playing games at home. This task of writing a diary was only assigned to the middle-aged adult group because the older adult group was not familiar with a web-based community.

### Data Collection and Analysis

#### Qualitative Analysis (RQ1 and RQ2)

For qualitative data entries, conversations during coplaying and discussion sessions were recorded and transcribed, including gaming diaries from middle-aged participants. The purpose of the qualitative analysis was to develop a practical mobile design guide for middle-aged and older adults in a holistic manner. The “customer journey map” suggested by service design studies [57] was utilized to include as many game elements as possible, offering contact points between users and gameplay in a coordinated series of processes from the start of the gameplay to the end.

First, we performed inductive thematic analysis on conversation transcripts and gameplay diaries to identify the positive and negative experiences of gameplay. We followed the 6 steps of thematic analysis proposed by Braun and Clarke [58] in this process. Thematic analysis is a useful method for summarizing the key features of a large data set and for gaining a rich understanding of people’s experiences [59]. To familiarize with the data, the first author read all the transcripts and diaries multiple times (step 1). Then, the first author generated initial codes (step 2) and developed these codes into themes of positive and negative experiences (step 3). Three authors reviewed and defined themes through discussion to reach a consensus (steps 4 and 5). All steps were repeated until a final version of the game design guides was acquired (step 6).

Second, we grouped themes of positive and negative experiences under the categories of elements related to gameplay. In order to define these categories, we first identified the basic elements (eg, audiovisuals, interface) by referring to the fundamental components presented by Ermi and Mäyrä [60]. Then, we added additional elements such as device, platforms, and sales, referring to the contents of a design document presented by Fullerton [61]. Finally, 10 elements were included: (1) cognitive and physical elements that are mainly addressed in existing user experience studies, such as audiovisual, interface, motor skill, and touch interaction; (2) psychological and socioemotional elements related to game content such as game rules, story and character, and social aspects; and (3) consumption contextual elements that are related to the game, such as devices, software installation, advertising, and in-game payments. We included consumption contextual elements in the guide because they also form an integrated user experience with core game elements.

Third, we generated design guides from each theme of experience and recommendation from the participants’ feedback. Three authors and one game design expert reviewed the themes and created proper guides. The final guides were compared with previous works to identify the guides that matched the previous works and the newly discovered guides.

#### Quantitative Analysis (RQ3)

For quantitative data, we requested survey responses regarding the satisfaction level with each element of the games played at the end of each session. The data were collected because the scores of the commercial games on each element can be a useful indicator for game designers. First, participants rated the satisfaction level of each game's elements on a 5-point scale for the game they played that day (1=very unsatisfied; 2=unsatisfied; 3=average; 4=satisfied; and 5=very satisfied). Table 4 shows the questionnaire regarding the satisfaction level with each game element. Five face emoticons were used for the 5-point scale to facilitate the answering process for participants (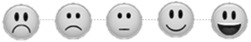).

Second, on the last day of the workshop, participants rated the overall satisfaction level of the game experience for each game on a 5-point scale. This allowed us to examine the satisfaction level when participants first encountered each game and the overall satisfaction after playing the game for a while.

**Table 4:** Survey questionnaires for measuring the satisfaction level.

Game element		Question
**Cognitive and physical elements**		
	Font size	Was the text legible with a reasonable font size?
	Button size	Was the size and location of the button appropriate?
	Sound	Were you satisfied with the sound of the game?
	Information amount	Was there an appropriate amount of on-screen information?
	Button interaction	Did you have any difficulty finding which button to press on each page?
	Speed	Was the game played at a proper speed to understand the content?
	Agility	Was it difficult when you needed a quick reaction? (eg, selecting moving targets, avoiding obstacles quickly)
**Psychological and socioemotional elements**		
	Objective	Was the goal to achieve in the game clear and appropriate?
	Resource/item	Was the method of using in-game resources and items clear and appropriate?
	Word comprehension	Did you understand the meaning of the words and sentences in the game?
	Mood/character/story	Did you like the atmosphere, character, and story of the game?
**Consumption contextual elements**		
	Setting to start	How was the setting process to start the game? (eg, loading the account, setting a nickname)
	Advertising	Were the in-game ads easy to deal with?
	Payment	Did you understand the in-game purchase system?

## Results

### Positive and Negative Experience With Gameplay (RQ1)

We identified 38 themes of positive experiences and 58 themes of negative experiences from dialogs, game diaries, and observations. The most frequently mentioned negative experience was difficulty in understanding the game rules. The second was frustration caused by repeated failures in action games because of lack of agility. The third was difficulty in distinguishing between important objects of similar color or design. Interestingly, the pleasure of playing together was found to be the most positive experience, followed by cognitive training. The third was sensational pleasure derived from special audiovisual effects such as all objects bursting at once. Based on the identified themes of positive and negative experiences, we proposed 45 mobile game design guides for the middle-aged and older adult population. For detailed lists of themes and guides, see the supplementary material (Multimedia Appendix 1).

### Mobile Game Design Guides for the Middle-Aged and Older Adult Population (RQ2)

#### Design Guides Related to Cognitive and Physical Elements

Table 5 shows the design guidelines related to cognitive and physical elements. We presented not only the proposed guides but also previous works that are consistent with our findings.

**Table 5:** Mobile game design guides on cognitive and physical elements.

Design guide (DG)				Previous works
**Audiovisual**				
	DG1	Design important object/character as distinguishable from others.	[20,22,36,62]	
	DG2	Provide voice dubbing when presenting stories or speech bubble.	[24]	
	DG3	Provide options to choose the size of the font and objects.	[20,22,24,25,36]	
	DG4	Avoid sounds that are too sharp or repetitive.	—^a^	
**Interface**				
	DG5	Present functions step-by-step rather than presenting excessive information in one screen.	[20,23]	
	DG6	Visually express functions of buttons for illiterate players.	[20,23]	
	DG7	Avoid multiple button controls at the same time unless they are essential to gameplay.	[20,62]	
	DG8	Provide user manual that explains the control and function of buttons.	[20]	
	DG9	Place important buttons in easy to find and touch locations.	[26]	
	DG10	Provide a mini-map.	—	
	DG11	Highlight touch area rather than suggesting to touch anywhere.	—	
	DG12	Provide a tutorial in case of repeated incorrect touch interactions.	—	
	DG13	Automatically confirm the termination of sliding actions after a specific time period.	—	
	DG14	Limit the area of operating touchpad on the screen.	—	
**Motor skills**				
	DG15	Provide practice session for beginners.	—	
	DG16	Provide hints for control timing when the player fails repeatedly.	—	
	DG17	Increase the process speed and difficulty incrementally.	[25]	
	DG18	Provide speed adjustment function.	[25]	

^a^Not available.

Several previous studies have reported design recommendations that consider age-related changes such as font size, interface, and motor skills in user interface design, including digital games [20,22-26,36,62]. We found similar experiences, especially in audiovisuals and interfaces. Negative experiences related to declining vision were most frequently identified. In addition, participants had negative experiences in complex interface designs that were cognitively burdensome.

Touch interaction in games is unique for older adult participants who lack gameplay experience and who are not familiar with sliding or swapping interactions. We developed design guides 11-14 based on the failure cases of touch interaction. Most failure cases were observed in older adult participants. We found that participants slid objects in the wrong direction or forgot to pull a finger away from the screen after the action. In addition, some participants mistakenly performed a tap action when a slide was required. Several participants failed to double-click because they tapped once or too slowly. Confusion among participants was aggravated when complex touch-interaction methods were required. The older adult group played “Lonely One: Hole-in-one,” in which a parabola appears when the player touches anywhere on the screen. The player should adjust the position of the parabola and release it, causing the object to move along the parabola. This interaction method is similar to the popular game “Angry Birds.” Participants often failed to draw a correct parabola because they touched the wrong starting point (too close to the edge of the screen). Moreover, they tried to draw a line with their finger along the parabolic dotted line. In addition, we found that participants felt uncomfortable when the speed of the moving character was too fast or too slow. We developed 4 guides regarding motor skills (design guides 15-18).

#### Design Guides Related to Psychological and Socioemotional Elements

Table 6 shows the design guidelines related to psychological and socioemotional elements. Similar to that reported in previous research [17,25,36,43], our participants easily adapted to a game that had clear goals and simple rules, but they became frustrated with a game that was difficult and insufficiently explained. They said they forgot the rules quickly over time, although they had learned how to play it. Therefore, we developed design guide 19 that offers sufficient explanation of the game rules [25] and design guide 20 that provides options for selecting the difficulty levels [25,36,43]. Furthermore, new design guides were developed from participants’ feedback, such as providing hints (design guide 21), tips during loading time (design guide 22), and notification messages to prevent resource abuse (design guide 23). In addition, many participants disliked the time limitation in a game; they felt nervous and stressed with the time limits. Therefore, we proposed design guide 25, “eliminate time limits or provide alternatives for level-passing,” which was also proposed in previous studies [20,36]. Some middle-aged participants were satisfied with restrictions on the play opportunities, such as limiting the number of hearts because it prevented them from becoming too addicted to games. We developed a design guide for the time management option, which was also proposed by De Schutter and Vanden Abeele [41].

In terms of story and character, participants expressed dissatisfaction with stories that were too simple or cliché. They were also dissatisfied with stories that lack sympathy or promoted negative emotions such as depression and gloom. Participants also criticized stereotypical expressions regarding the lives of older adults. Instead, participants liked game stories that appealed to and were empathetic to players, stimulated their curiosity, and provided useful knowledge. One participant felt satisfied when another character in the game praised the player. Similar guides and recommendations were reported from previous works [10,36,43]; however, a new guide was proposed regarding a variety of interactive storytelling options such that the player can reflect their own preference (design guide 30). We found many positive effects of gameplay from participants’ feedback and identified those experiences to themes of positive experience under the category of affective aspects: feeling of fun and flow, cognitive skill training, sense of achievement, strategic thinking, concentration, learning something new, killing time, connecting stories to real life experiences and finding meaning, and reminiscing.

Similar to previous studies, we found that many older adult participants perceived gameplay as cognitive training activities [35]. For example, they mentioned “It was good because I feel like [I am] training my brain,” “It is likely to prevent dementia,” and “I feel like I'm getting smarter because I keep using my brain.” Moreover, participants said that the perception of beneficial effects is an important motivation to play. De Carvalho et al [36] also highlighted that clarifying the benefits of the game for older adults is important. We added design guide 31, which informs the affective aspect and beneficial effects of the game such as cognitive training, strategic thinking, learning, connecting real life, and reminiscing. We found that the results were consistent with previous studies on social dimensions [24,41,43]; participants said they had more fun playing with others than when playing alone. They were especially satisfied that they were able to accomplish a difficult mission with supporters on their side. They also felt good when they worked together with an unknown player whom they met in the game to complete the mission. However, some middle-aged participants found it harder to play games with their children because they had different preferences and gameplay skills.

**Table 6:** Mobile game design guides on psychological and socioemotional elements.

Design guide (DG)			Previous works
**Game rules**			
	DG19	Provide guidance, tutorials, and practice sessions.	[25]
	DG20	Provide options for selecting difficulty levels.	[25,36,43]
	DG21	Provide hints when the player fails repeatedly or is taking too much time.	—^a^
	DG22	Provide game rules and tips during loading time (repetitive tutorials).	—
	DG23	Provide a notification message if players abuse resources early in the game.	—
	DG24	Provide an appropriate challenge rather than a simple or easy rule.	[36,42]
	DG25	Eliminate time limits or provide alternatives for level-passing.	[20,36]
	DG26	Provide time management options.	[41]
**Story and character**			
	DG27	Provide familiar languages and concepts to the player (eg, based on culture and age).	[20,26]
	DG28	Provide players with complimentary messages or motivational feedback.	[10,36,43]
	DG29	Do not indicate life of older adults in static, passive, negative, and depressed tones.	[63]
	DG30	Provide options for players to choose stories and reflect diversity.	—
**Affective aspect and perceived benefit**			
	DG31	Inform players of the affective aspect and beneficial effects of the game (eg, cognitive training, strategic thinking, learning, connecting real life, reminiscing)	[36]
**Social aspect**			
	DG32	Consider using multiplayer mode or coplaying context in single play mode.	[24,41,43]
	DG33	Remove chat features in competitive games or only allow consensual chat between players.	—
	DG34	Restrict the use of abusive language.	—

^a^Not available.

One middle-aged participant was rather satisfied with the lack of chat function when playing “Brawl Stars” because she never heard swear words or slang from other players. Alternatively, it was less burdensome if the chat function was set to be available after establishing some acquaintances. However, participants were uncomfortable when the method for connecting to social media or inviting friends was complicated. Some participants were concerned about other people; they were worried about bothering others while turning the sound on or exhibiting excessive action during gameplay. One participant said that she did not want to connect to social media and show others that she played games because there were many formal relationships on social media.

#### Design Guides Related to Consumption Contextual Elements

Table 7 shows the design guidelines related to consumption contextual elements, such as devices, software installation, and advertising/payment. Most guides under this category are newly written. We found that older adults preferred using tablet personal computers rather than mobile phones because of their deteriorating eyesight resulting from aging. However, using tablet personal computers was not comfortable when participants unintentionally touched the screen. Furthermore, because of the apparent lack of moisture on their fingers, touch recognition did not work well for some of the participants. We provided additional equipment such as touch pens and controllers; touch pens worked well with games where small objects were to be touched. Similarly, middle-aged adults preferred using a specially developed controller while playing “Brawl Stars.” Therefore, encouraging the use of appropriate devices and additional equipment for middle-aged and older adults may be useful. Installing game software on devices is a challenge for some older adult participants who lack experience in using smartphones. They had difficulty typing and searching for apps. However, they became accustomed to it through repeated practice. Participants said they felt confused with different games having similar titles. Many older adult participants could not remember their IDs and password. Without help, it was difficult for them to register for or log into a game. This issue has been reflected in design guide 38, but special care is needed to prevent misuse of personal information. In contrast, middle-aged adults did not have much trouble with their installation. However, waiting for large files to download was boring for some participants and they did not want to pay for data usage to download games.

Participants felt uncomfortable and bored when there were too many ads or when the ads were too long. In addition, they felt uncomfortable when they are suddenly required to watch an ad while playing because it interrupts their game flow. They were upset that advertisements with inappropriate content such as adult content suddenly appear sometimes without any notification. Participants sometimes became confused whether the pop-up interaction was an advertisement or the game they were playing. They were also confused because they did not know how to turn the ads off, especially when the clickable “close” button feature was too small or when there was a countdown before the appearance of the close button. Participants hoped to be provided with items, hints, and other benefits of gameplay when watching advertisements. Some older adult participants were very generous regarding advertisements. They said that it was okay because they could take a short break and were provided new information during the advertisement.

Regarding payment, older adult participants had no intention of purchasing games or items. They did not connect their credit card to the app store because they were afraid of being mischarged. However, some middle-aged adults paid to buy items to gain an advantage in the game. Both groups were offended when phrases inducing such charges appeared at a difficult level.

**Table 7:** Mobile game design guides on consumption contextual elements.

Design guide (DG)			Description
**Devices**			
	DG35	Able to play both in a tablet personal computer and mobile phone.	
	DG36	Provide supportive equipment (eg, touch pen, controller).	
**Installation and setting to start**			
	DG37	Inform expected install time and file sizes.	
	DG38	Load account information automatically with privacy precautions.	
	DG39	Use easy and unique game titles, which do not overlap with other games.	
**Advertisement and payment**			
	DG40	Provide items and hints after viewing ads.	
	DG41	Avoid excessive or long ads, which interrupt gaming.	
	DG42	Make it easy to turn off the ads.	
	DG43	Present suitable ads for ages and preference (avoid inappropriate content).	
	DG44	Let the player choose the timing for advertisements (avoid sudden ads that interrupt the game flow).	
	DG45	Noninteractive ads are preferred over interactive ads.	

### Satisfaction Level in Each Element of the Games (RQ3)

**Figure 2:**
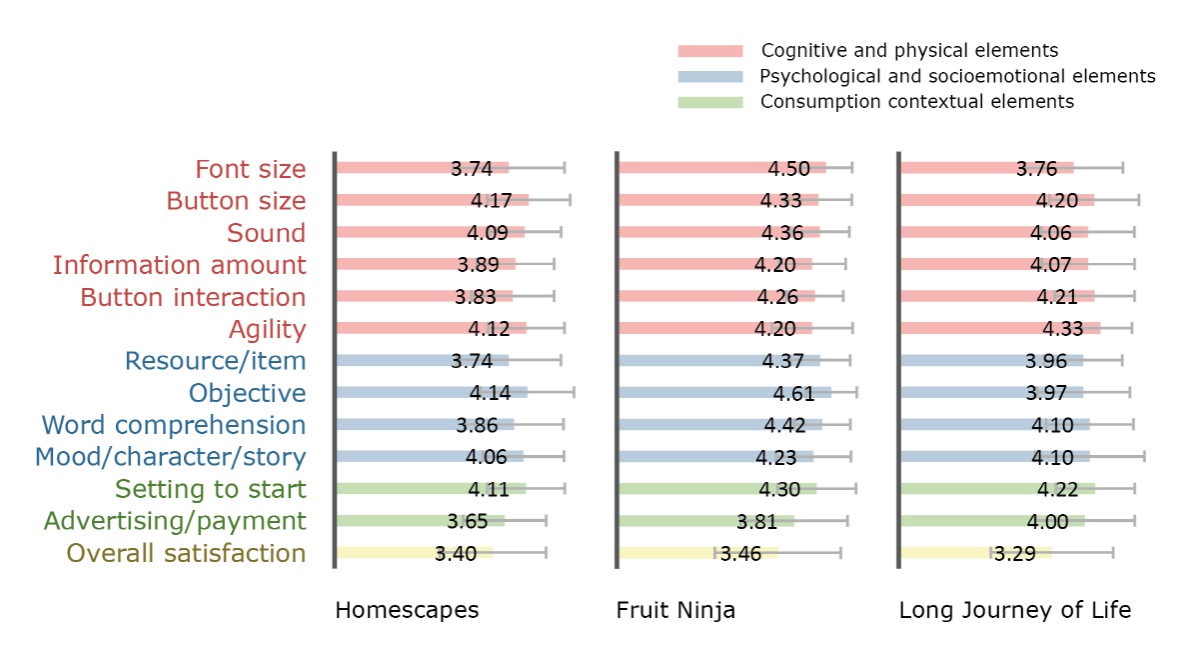
Satisfaction levels in games played by both groups.

**Figure 3:**
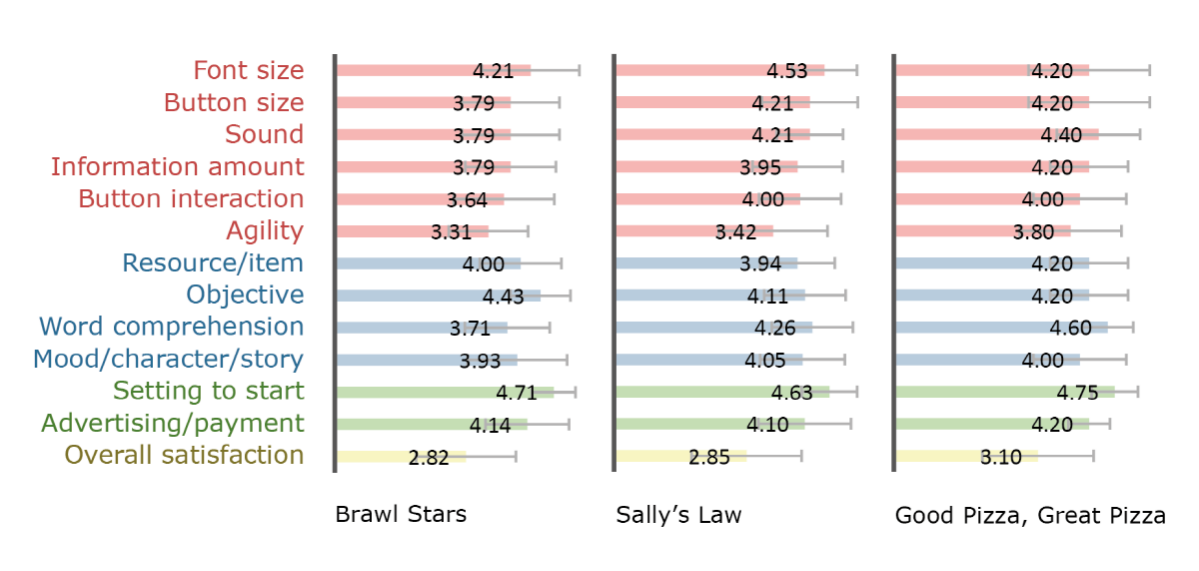
Satisfaction levels in games played by the middle-aged group.

**Figure 4:**
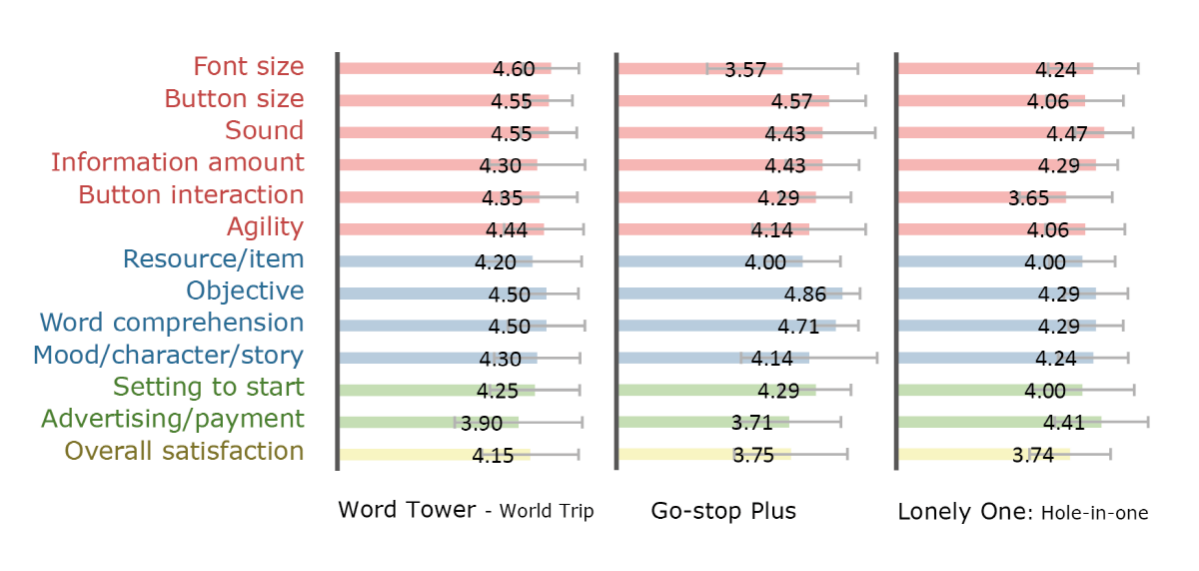
Satisfaction levels in games played by the older adult group.

Figures 2-4 show participants’ satisfaction level for each game’s elements and overall satisfaction on the 5-point scale. The goal of this descriptive statistics is to provide game designers with a visual profile of how players are satisfied with game elements in the game case. The satisfaction level for each game’s elements was measured in each session; therefore, they were treated as first impressions after the first 30 minutes of gameplay. The overall satisfaction level in each game was measured on the last day of the workshops. This score is a satisfaction evaluation by comparing it to other games after several days of gameplay. Overall, we can observe from the figures that the satisfaction level with the individual elements performed on each session tended to be higher than the overall satisfaction evaluated on the last day of the workshop. For additional information on Figures 2-4 such as the mean and standard deviation (SD) of the satisfaction levels in each game, see the supplementary material (Multimedia Appendix 2).

Regarding games played by both groups (Figure 2), the mean scores of “Fruit Ninja” in all game elements were over 4.0, except for advertising/payment, but the overall satisfaction was only 3.46 (SD 1.35). It is assumed that even though this game was intuitive and easy to use, there were wide differences in the overall preference between the participants. The scores of “font size” were a bit lower on “Homescapes” and “Long Journey of Life” than those of other elements; we found that many participants mentioned that the text is too small in those games.

Regarding games played by the middle-aged group only (Figure 3), action games such as “Brawl Stars” and “Sally’s law” were found to be less satisfactory with agility than other elements, and the overall satisfaction was low. The 2 games seem to provide participants with too much difficulty in terms of agility. In addition, the button interaction of “Brawl Stars” was also low (mean 3.64 [SD 1.08]), which seems to have been difficult for participants to control buttons simultaneously using both hands.

Regarding games played by the older adult group only (Figure 4), the mean scores of “Word Tower-World Trip” were estimated to be over 4.0 in almost all elements, with a high overall satisfaction level (mean 4.15 [SD 1.04]). Scores of “objective” and “word comprehension” in “Go-stop Plus” were high because it was a digital version of the traditional Korean card game popular with older adults. However, font size was less satisfactory than other elements in this game. Regarding “button interaction,” “Lonely One: Hole-in-one” scored only 3.65 (SD 1.00), apparently because adjusting the position of the parabola to determine the direction of the ball’s flight was unfamiliar to older adults.

### Timeline of the Study

This project was funded from April 2019 to December 2021 and approved by the institutional review board on June 7, 2019. The data were collected at the workshops involving 40 middle-aged and older adults and 11 young partners from July 1, 2019 to August 28, 2019.

## Discussion

### Overview of This Study

The purpose of this study was to derive mobile game design guidelines for the middle-aged and older adult population by analyzing user experience through a qualitative thematic analysis. We proposed 45 guides under the framework of 10 game elements. In addition, we reported descriptive statistics showing the satisfaction profiles for 9 game cases. Based on these results, we had the following discussion.

### Embrace the Middle-Aged and Older Population: Reaffirmed Guides and Newly Discovered Guides

Previous studies reported that aging issues such as decreased eyesight, memory, and motor skills could lead to difficulties in playing digital games targeted at the younger generation [15,16,18]. Game design guides for older adults should focus on age-related changes and game elements [22-25]. This study reaffirmed these previous issues and noted that these guidelines have not been applied to many commercial mobile games. We rediscovered repeatedly reported issues such as the size of the text and objects, cognitive burden at the interface, and agility problems. These confirmed guidelines should be essential considerations for game designers who want to create games that cater to the middle-aged and older adult population. In previous studies, it was rare to observe all contact points from the beginning to the end of the mobile gaming experience. By including the context of game consumption, this study was able to introduce new guidelines. These are the parts related to the unique touch interaction used in the game and the parts related to the use of the app store and advertising/payment features embedded in the gaming experience.

### Prior Experience of Computer and Digital Games

The use of new technologies is more likely to be a response to historical changes in the older generation than age-related declines [64]. We also found that the prior experience in using digital devices and games affects the participants’ overall gameplay. This is because many digital games require basic digital skills such as installation, typing, double-clicking, and cancellation. While middle-aged participants who are already experienced in the use of computers and smartphones had no problems with installation, typing, and various touch-based interactions, older adult participants needed more help to familiarize themselves with basic operations. In addition, many games use the design elements of earlier successful games. For example, gamers are familiar with a heart-shaped icon that implies remaining opportunities. Moreover, digital games of the same genre often use similar interaction methods. Therefore, compared to the younger generation who are unconsciously trained on these norms through various games since childhood, older adults, who lack gameplay experience, need time to adapt to a new game. Because of these factors, participants recognized gameplay as a learning activity rather than a leisure activity. Charlier et al [65] stressed that older adults’ experiences may include more traditional learning and less game-based learning. Our participants wanted to read specific manuals or learn from others before they started playing. In addition, participants mentioned that they had to recognize the benefits of a game and how it helped them start the gameplay. Therefore, when encouraging older adults to play games, it will be necessary to persuade them to show them how these games are beneficial to them [34,36,37]. In addition, it is recommended to provide game literacy education programs, which introduce the benefits of games, recommend proper games for them, and explain how to play games.

### Game Aesthetics and Socioemotional Aspects for the Middle-Aged and Older Adult Population

According to the mechanics, dynamics, and aesthetics framework proposed by Hunicke et al [66], aesthetics refers to the emotions that the player experiences while playing the game. The game designer wants to arouse certain emotional responses in the player, such as sensation, fantasy, narrative, challenge, fellowship, discovery, expression, and submission. De Schutter [67] extended the list to include older adults’ gaming experience and presented 6 items as “Geronto-Aesthetics,” such as cultivation, compensation, connectedness, contribution, nostalgia, and contemporaneity. Through our empirical research, we discovered themes consistent with those in previous studies: challenge (sense of achievement, strategic thinking, and concentration), sensation (feeling of fun and flow), discovery (learning something new), submission (killing time), cultivation (connecting stories to real life experiences and finding meaning), and nostalgia (reminiscing). Moreover, we identified one more item, that is, cognitive skill training. Gameplay is a meaningful activity for older adults because they are interested in cognitive training and in mitigating dementia [35].

Regarding the social aspect of the gaming experience, participants enjoyed playing games together [38,39] and they positively evaluated the interactions with the younger generation. In this study, playing together does not necessarily mean solely playing multiplayer games. Various social interactions could also occur during games that offer only single-player options, for example, turn-taking, score competition, helping with difficult parts, and vicarious play. Young adult partners acted as supporters and coplayers during the workshop; without this help, it would have been even more difficult for the older population to use the game alone. In addition, participants preferred cultural content that they were familiar with, and many women were reluctant to engage with violent content [16].

### Limitations and Future Works

There are several limitations associated with this study and a number of issues that remain unexplored. First, a limited number of games were played because of the short workshop duration. Users’ personal preference and styles are important factors, but we did not reflect them when we chose the games. Depending on the diverse interests of middle-aged and older adults, we need to discover and expose them to more games suiting their preferences.

Second, extra caution is required to generalize the results because we could not recruit a more diverse participant pool. All the participants were Koreans, and the results could vary depending on participants from different cultural backgrounds and technology dissemination. In addition, we could not recruit participants who are prescribed or recommended gaming but who have no affinity to games. Moreover, as more female participants were recruited, the study does not include plentiful experiences of male participants. However, since mobile games are especially popular with middle-aged and older women [3], their experiences might be meaningful for mobile game designers.

Third, special attention is required when interpreting the results because the presence of young adults in the workshop setting could impact the gaming experience of participants. Our participants were able to obtain immediate help during the workshop from young partners; however, in many cases, playing alone would likely cause more difficulties.

Fourth, the scope of the quantitative analysis reports descriptive statistics at the exploration stage. The purpose of the quantitative analysis was to provide a useful indicator to game designers by showing the visual profiles of each case. Therefore, these tentative results require further refinement in strictly controlled experiments.

Lastly, with regard to the prior experience with computers and digital games, we found that there were differences between middle-aged adults and older adults in terms of gaming experience, but further research is needed to clarify these differences. In addition, when the digital native generation in their 20s and 30s is older, this guide is likely to have different criteria. Technologies are gradually evolving to be available intuitively without special learning. Therefore, this guide will require continuous updates through user studies from different cohorts.

### Conclusion

This study includes considerations when creating games for the middle-aged and older population. Game design guides were produced based on the feedback of middle-aged and older adults during the game workshops, where they played mobile games of various genres. The implications of this study are suggesting a design guideline focusing on 10 categories of game elements. Consequently, game designers can holistically understand the game experience of older adults and easily find the relevant information. In addition, our empirical research was able to reaffirm the proposals from previous works that sought to improve the usability of a user interface. Furthermore, we also provided new guides for game design, such as touch interaction, game rules, stories, and advertising/payments. We found that most elements that participants were uncomfortable with during gameplay could be applied to all generations, regardless of age. The young generation also experiences difficulties with using touchscreen technologies, but they are better at adopting technological changes [68]. Thus, improving older adults’ gaming experience may improve the experience for other generation groups as well. Rather than having special game design rules for the older population, we found that a universal design that can be easily accessed by gaming novices irrespective of their generation, is more important. In addition, design guides targeting specific capabilities and limitations are not effective because the older population is diverse in its ranges and combinations of limitations [69]. We hope this guide will be useful for various activities related to gameplay for the middle-aged and older adult population, such as for developing games targeting this population, modifying existing games while considering accessibility, and developing game-related educational programs for this population.

## Appendix

Multimedia Appendix 1 Mobile game design guides for the middle-aged and older adult population.

Multimedia Appendix 2 Descriptive statistics of participants’ satisfaction levels in each game case.

## Abbreviations

RQ: research question
