# Supplementary material for: Mobile Game Design Guide to Improve Gaming Experience for the Middle-Aged and Older Adult Population: User-Centered Design Approach
Source: JMIR Serious Games. 2021 May 20;9(2):e24449. doi: 10.2196/24449 (PMC8176340; doi:10.2196/24449)
Supplement: Multimedia Appendix 1 [file games_v9i2e24449_app1.pdf]

Multimedia Appendix 1

Mobile Game Design Guide

for the Middle-Aged and Older Adult Population

●PE: Positive Experience
●NE: Negative Experience
●DG: Design Guide

1. Cognitive and physical elements

Audiovisuals	
●PE1	Stress relieved with special effect (eg, objects cleared at once)
●PE2	Adequate size of text and objects
●PE3	Satisfied with design (eg, bright atmosphere, various colors, detail)
●PE4	Satisfied with sound (eg, harmony with visuals, comfortable)
●NE1	Difficult to recognize important objects (eg, confused with similar colors)
●NE2	Too small size of texts and objects
●NE3	Dissatisfied with design (eg, too simple, cliché, not familiar)
●NE4	Dissatisfied with sound (eg, too loud, constant repeat)
●DG1	Design important object/ character as distinguishable from others
●DG2	Provide voice dubbing when presenting stories or speech bubble
●DG3	Provide options to choose the size of the font and objects
●DG4	Avoid sounds that are too sharp or repetitive

Motor skills	
●PE8	Felt thrill, excitement after success of agility/ timing challenges
●PE9	Adequate speed of screen switch or character moving
●NE21	Too difficult levels of agility and timing
●NE22	Too fast or slow speed of screen switching or motion of characters
●NE23	Physical fatigue when playing games for a long time
●NE24	Non-preference for games that require agility
●DG15	Provide practice session for beginners
●DG16	Provide hints for control timing when the player fails repeatedly
●DG17	Increase the process speed and difficulty incrementally
●DG18	Provide speed adjustment function

Interface	
●PE5	Easy to find button
●PE6	Adequate information on one screen
●PE7	Visually well-expressed buttons for functions
●NE5	Need multiple button controls for moving and interacting
●NE6	Not enough tutorial for button functions
●NE7	Excessive information on one screen
●NE8	Unable to read a story (eg, story presented during actions)
●NE9	Tough to find button position (eg, located in the corners)
●NE10	Unable to find locations of self/opponent characters
●NE11	If touches are available over whole screen – Not sure where to touch
●NE12	Difficult to realize one's turn in a turn-based game
●NE13	Repeated touch slides with fingers rubbing
●NE14	Sliding in the wrong direction or at the wrong time
●NE15	Pressing buttons too hard during action games when tensed up
●NE16	Difficulty with connecting objects by sliding interaction
●NE17	Difficulty with using direction aiming lines (eg, attempts to slide along a line)
●NE18	Attempting taps when sliding is required
●NE19	Difficulty with double click (tap) interactions
●NE20	Touch errors (eg, touch with wrist)
●DG5	Present functions step-by-step rather than presenting excessive information in one screen
●DG6	Visually express functions of buttons for illiterate Players
●DG7	Avoid multiple button control at the same time unless they are essential to game play
●DG8	Provide user manual that explains control and functions of buttons
●DG9	Place important buttons in easy to find and touch locations
●DG10	Provide a mini-map
●DG11	Highlight touch area rather than suggesting to touch anywhere
●DG12	Provide a tutorial in case of repeated incorrect touch interactions
●DG13	Automatically confirm the termination of sliding actions after a specific time period
●DG14	Limit the area of operating touchpad on the screen

2. Psychological and socioemotional elements

Game rules

●	PE10	Clear goals, easy and simple rules
●	PE11	Interesting functions (eg, combo, revival, hiding, collecting)
●	PE12	Hints provided at the right time
●	PE13	Relaxed with no time limit
●	PE14	Limit chances to play because it prevents game addiction
●	PE15	Providing various modes and options
●	PE16	Auto-play mode
●	PE17	Nostalgia for similar games in the past
●	PE18	Online version of traditional games
●	NE25	Forgetting gaming methods
●	NE26	Difficult to understand the use of resource/items (eg, wasting important resources)
●	NE27	Difficult rules with a lack of explanation
●	NE28	Unstable level of difficulties (too hard or too easy)
●	NE29	Difficult to accept new type of games (having a stereotype about games)
●	NE30	Unsatisfied rules
●	NE31	Became nervous and stressed because of the time limit
●	NE32	Regret due to excessive gameplay
●	NE33	Difficult to understand foreign languages related to game guidance
●	DG19	Provide a guidance, tutorial, and practice sessions
●	DG20	Provide options for selecting difficulty levels
●	DG21	Provide hints when the player fails repeatedly or is taking too much time
●	DG22	Provide game rules and tips during loading time (repetitive tutorials)
●	DG23	Provide a notification message if players abuse resources early in the game
●	DG24	Provide an appropriate challenge rather than an extremely simple or easy rule
●	DG25	Eliminate time limits or provide alternatives for level-passing
●	DG26	Provide time management options

Affective aspect and perceived benefit

●	PE22	Cognitive skill training
●	PE23	Sense of achievement
●	PE24	Strategic thinking
●	PE25	Concentration
●	PE26	Feelings of fun and flow
●	PE27	Learning something new
●	PE28	Killing time and relieving stress
●	PE29	Connecting stories to real life experiences and finding meaning
●	PE30	Reminiscing
●	DG31	Inform players of affective aspects and beneficial effects of the game (cognitive training, strategic thinking, learning, connecting real life, reminiscing, etc.)

Story and character

●	PE19	Satisfied with stories (eg, touching, curious, exciting)
●	PE20	Likable character (eg, familiar look, attractive personality)
●	PE21	Satisfied when the player is praised by game characters
●	NE34	Unable to understand the meaning of the story (eg, lack of explanation, wrong translation)
●	NE35	Dissatisfied with the story (eg, too simple, cliché, depressed, lack of empathy)
●	NE36	Dislike characters (eg, childish, unlikable personality)
●	NE37	Dislike the violent elements
●	DG27	Provide familiar languages and concepts to the player (eg, based on culture and age)
●	DG28	Provide players with complimentary messages or motivational feedback
●	DG29	Do not indicate life of older adults in static, passive, negative, and depressed tones
●	DG30	Provide options for players to choose stories and reflect diversity

Social aspect

●	PE31	More enjoyable when playing games together
●	PE32	Side-by-side help from supporters
●	PE33	Mission accomplished with help or cooperation from other players
●	PE34	Satisfied with no chatting function because no profanity/slang from other players
●	NE38	Difficult to understand how to invite friends
●	NE39	Afraid of being a burden to other players
●	NE40	Wanting to play with children, but finding it difficult because of a generation gap in preference and game skills
●	NE41	Afraid of bothering other people (eg, exposing excessive gaming action, not willing to connect to SNS)
●	DG32	Consider using multiplayer mode or co-playing context in single play mode
●	DG33	Remove chat features in competitive games or only allow consensual chat between players.
●	DG34	Restrict the use of abusive language

3. Consumption contextual elements

Devices	
●PE35	Satisfied with screen size of the tablet PC, which is bigger than cellphone
●PE36	Satisfied with using assisting tools (eg, touch pen, controller etc.)
●NE42	Touch failure (eg, not enough moisture on the finger)
●NE43	Dissatisfied with small screen with cellphone
●NE44	Inconvenience with assisting tools
●NE45	Device failed to recognize when entering foreign language
●DG35	Able to play both in a tablet PC and mobile phone
●DG36	Provide supportive equipment (eg, touch pen, joystick etc.)

Installation and setting to start	
●NE46	Long installation time/Heavy data usage
●NE47	Difficulty with linking to SNS accounts
●NE48	Difficulty using Appstore (eg, search and download)
●NE49	Not familiar with utilizing various applications
●NE50	Difficulty with typing with the keyboard
●DG37	Inform expected install time and file sizes
●DG38	Load account information automatically with privacy precautions
●DG39	Use easy and unique game titles, which do not overlap with other games

Advertising and payment	
●PE37	Items provided after watching ads
●PE38	Ads providing new information and giving a short break
●NE51	Too many and too long ads
●NE52	Difficult to turn off the ads (very small clickable "close" button, countdown, etc.)
●NE53	Ads with inappropriate content (eg, adult content)
●NE54	Being forced to watch ads
●NE55	Inducing payments to pass at the difficult game level (Presenting phrases that induces payment excessively)
●NE56	Not comfortable with interactive ads because it creates confusion between the ads and the game
●NE57	Difficult to understand complex cash payment system
●NE58	Afraid of being mischarged
●DG40	Provide items and hints after viewing ads
●DG41	Avoid excessive or long ads, which interrupt gaming
●DG42	Make it easy to turn off the ads
●DG43	Present suitable ads for ages and preference (avoid inappropriate content)
●DG44	Let the player choose the timing for advertisements (avoid sudden ads that interrupt the game flow)
●DG45	Non-interactive ads are preferred over interactive ads
