# Supplementary material for: Mobile Game Design Guide to Improve Gaming Experience for the Middle-Aged and Older Adult Population: User-Centered Design Approach
Source: JMIR Serious Games. 2021 May 20;9(2):e24449. doi: 10.2196/24449 (PMC8176340; doi:10.2196/24449)
Supplement: Multimedia Appendix 2 [file games_v9i2e24449_app2.pdf]

Multimedia Appendix 2: Descriptive statistics of participants' satisfaction level in each game case

1. Games played by both groups (Figure 2)

	Game case					
	Homescapes		Fruit Ninja		Long Journey of Life	
**Game elements**	M (SD)	n	M (SD)	n	M (SD)	n
Font size	3.74 (1.20)	35	4.50 (0.56)	36	3.76 (1.06)	29
Button size	4.17 (0.89)	35	4.33 (0.72)	36	4.20 (0.96)	30
Sound	4.09 (0.78)	35	4.36 (0.64)	36	4.06 (1.00)	31
Information amount	3.89 (0.83)	35	4.20 (0.72)	35	4.07 (1.00)	29
Button interaction	3.83 (0.89)	35	4.26 (0.61)	35	4.21 (0.86)	29
Agility	4.12 (0.82)	33	4.20 (0.87)	35	4.33 (0.68)	27
Resource/item	3.74 (1.12)	35	4.37 (0.65)	35	3.96 (0.84)	28
Objective	4.14 (1.00)	35	4.61 (0.55)	36	3.97 (1.00)	30
Word comprehension	3.86 (1.06)	35	4.42 (0.60)	36	4.10 (0.94)	29
Mood/character/ story	4.06 (0.87)	35	4.23 (0.81)	35	4.10 (1.18)	29
Setting to start	4.11 (0.83)	35	4.30 (0.85)	37	4.22 (0.85)	27
Advertising/payment	3.65 (0.89)	26	3.81 (1.15)	32	4.00 (1.07)	15
Overall satisfaction	3.40(1.14)	35	3.46 (1.35)	39	3.29 (1.31)	34

2. Games played by middle-aged group (Figure 3)

	Game case					
	Brawl Stars		Sally's Law		Good Pizza, Great Pizza	
**Game elements**	M (SD)	n	M (SD)	n	M (SD)	n
Font size	4.21 (1.05)	14	4.53 (0.70)	19	4.20 (1.30)	5
Button size	3.79 (1.05)	14	4.21 (1.03)	19	4.20 (1.30)	5
Sound	3.79 (1.05)	14	4.21 (0.71)	19	4.40 (0.89)	5
Information amount	3.79 (0.97)	14	3.95 (0.97)	19	4.20 (0.84)	5
Button interaction	3.64 (1.08)	14	4.00 (0.88)	19	4.00 (1.00)	5
Agility	3.31 (0.85)	13	3.42 (1.17)	19	3.80 (1.10)	5
Resource/item	4.00 (0.88)	14	3.94 (0.80)	18	4.20 (0.84)	5
Objective	4.43 (0.65)	14	4.11 (0.88)	19	4.20 (0.84)	5
Word comprehension	3.71 (0.91)	14	4.26 (0.87)	19	4.60 (0.55)	5
Mood/character/story	3.93 (1.07)	14	4.05 (0.91)	19	4.00 (1.00)	5
Setting to start	4.71 (0.47)	14	4.63 (0.60)	19	4.75 (0.50)	4
Advertising/payment	4.14 (0.90)	7	4.10 (0.99)	10	4.20 (0.45)	5
Overall satisfaction	2.82 (1.07)	17	2.85 (1.18)	20	3.10 (1.20)	10

3. Games played by older adult group (Figure 4)

	Game case					
	Word Tower - World Trip		Go-stop Plus		Lonely One: Hole-in-one	
**Game elements**	M (SD)	n	M (SD)	n	M (SD)	n
Font size	4.60 (0.60)	20	3.57 (1.62)	7	4.24 (0.97)	17
Button size	4.55 (0.51)	20	4.57 (0.79)	7	4.06 (0.83)	17
Sound	4.55 (0.60)	20	4.43 (1.13)	7	4.47 (0.62)	17
Information amount	4.30 (1.03)	20	4.43 (0.79)	7	4.29 (0.47)	17
Button interaction	4.35 (0.81)	20	4.29 (0.76)	7	3.65 (1.00)	17
Agility	4.44 (0.86)	18	4.14 (1.21)	7	4.06 (0.85)	16
Resource/item	4.20 (1.06)	20	4.00 (0.82)	7	4.00 (0.71)	17
Objective	4.50 (0.69)	20	4.86 (0.38)	7	4.29 (0.69)	17
Word comprehension	4.50 (0.83)	20	4.71 (0.49)	7	4.29 (0.59)	17
Mood/character/story	4.30 (0.92)	20	4.14 (1.46)	7	4.24 (0.75)	17
Setting to start	4.25 (0.97)	20	4.29 (0.76)	7	4.00 (1.12)	17
Advertising/payment	3.90 (1.37)	20	3.71 (1.11)	7	4.41 (1.00)	17
Overall satisfaction	4.15 (1.04)	20	3.75 (1.22)	12	3.74 (0.87)	19
